# Supplementary material for: High-resolution single-cell 3D-models of chromatin ensembles during Drosophila embryogenesis
Source: Nat Commun. 2021 Jan 8;12:205. doi: 10.1038/s41467-020-20490-9 (PMC7794469; doi:10.1038/s41467-020-20490-9)
Supplement: Supplementary file 1 — Supplementary Information [file 41467_2020_20490_MOESM1_ESM.pdf]

High-Resolution Single-Cell 3D-Models of Chromatin Ensembles during Drosophila Embryogenesis

Qiu Sun[†1], Alan Perez-Rathke[†2], Daniel M. Czajkowsky[3], Zhifeng Shao[*3] and Jie Liang[*2]

[1] *Shanghai Center for System Biomedicine , Shanghai Jiao Tong University, Shanghai 200240, China*

[2] *Department of Bioengineering, University of Illinois at Chicago, SEO, MC-063, Chicago, IL 60607-7052, USA*

[3] *School of Biomedical Engineering, Shanghai Jiao Tong University, Shanghai 200240, China*

[†] These two authors contributed equally.

[*] Corresponding authors: zfshao@sjtu.edu.cn to ZS and jliang@uic.edu to JL

Supplementary Figures

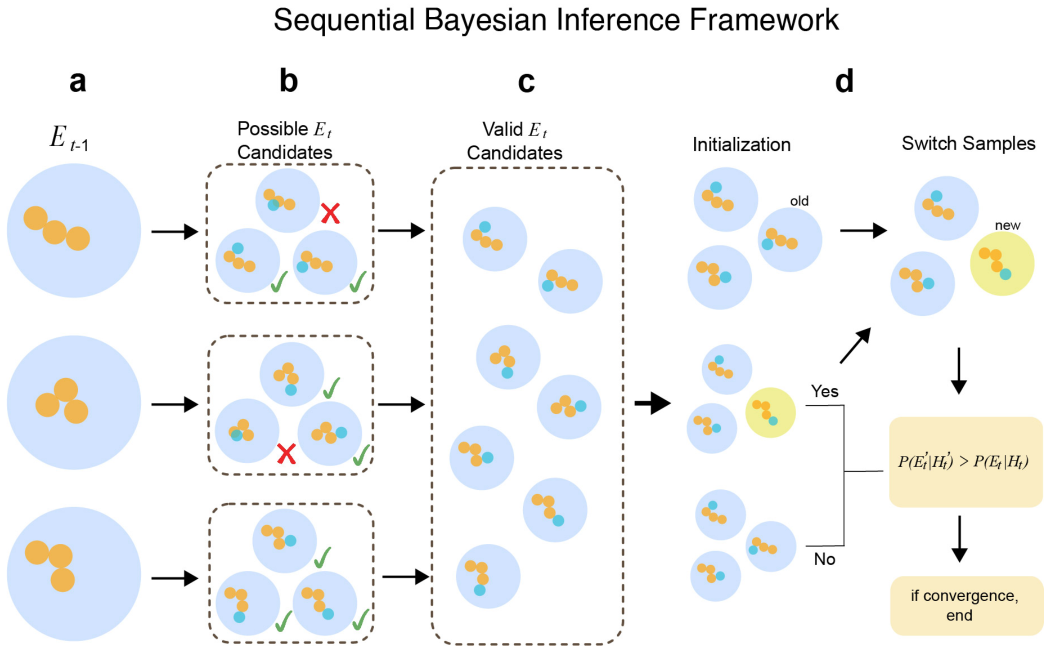

Supplementary Figure 1. Overview of the sequential Bayesian inference framework. **(a)** For each configuration $X_{t-1}^{(k)}$ in the ensemble E_{t-1}, **(b)** we list all possible configuration candidates $X_t^{(k)}$ with newly placed bead $x_t^{(k)}$, then filter out the candidates that violate the physical constraints of self-avoiding property, **(c)** followed by pooling all valid X_t candidates together to generate our sampling space. **(d)** We first pick an initial polymer ensemble E_t from the sampling pool and compute the Bayesian probability $P\left(E_t|H_t\right)$, then randomly switch one polymer chain in the ensemble to another valid candidate and compute the new probability $P\left(E_t'|H_t'\right)$, we keep the new polymer chain in the ensemble if $P\left(E_t'|H_t'\right) > P\left(E_t|H_t\right)$. This optimization program will end until convergence.

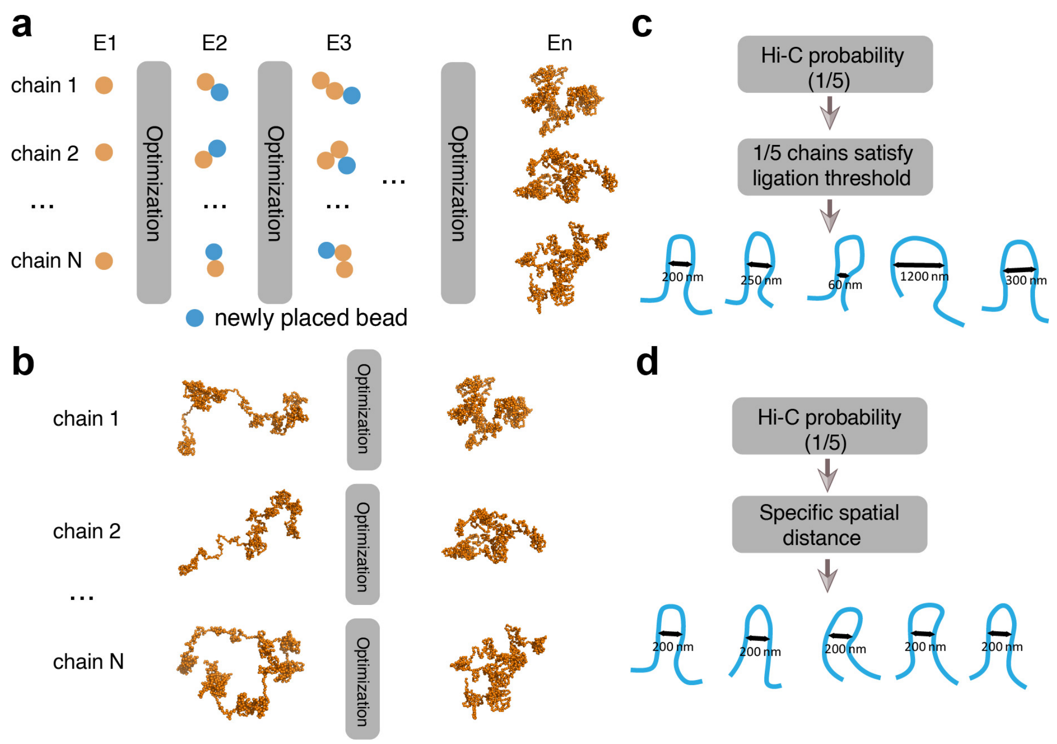

Supplementary Figure 2. An illustration of our method based on chain growth and comparison with other methods. **(a)** Chromatin polymer ensemble of 50,000 is constructed sequentially, with the addition of one bead at a time. We make use of an optimized sampling distribution at each step to improve sampling efficiency, which is dynamically adjusted after adding each bead. **(b)** Construction of chromatin polymer ensemble through two other data-driven approaches: MOGEN [19] and the method of Wang et al [12]. All beads are placed at once during initialization and each chromatin chain is subject to a separate optimization procedure where the object function is based on each single chain. **(c)** In our model, Hi-C probability corresponds to the proportion of polymer chains that satisfy the ligation threshold. **(d)** In other methods, a Hi-C probability is transformed to certain spatial distance via a reverse function [12].

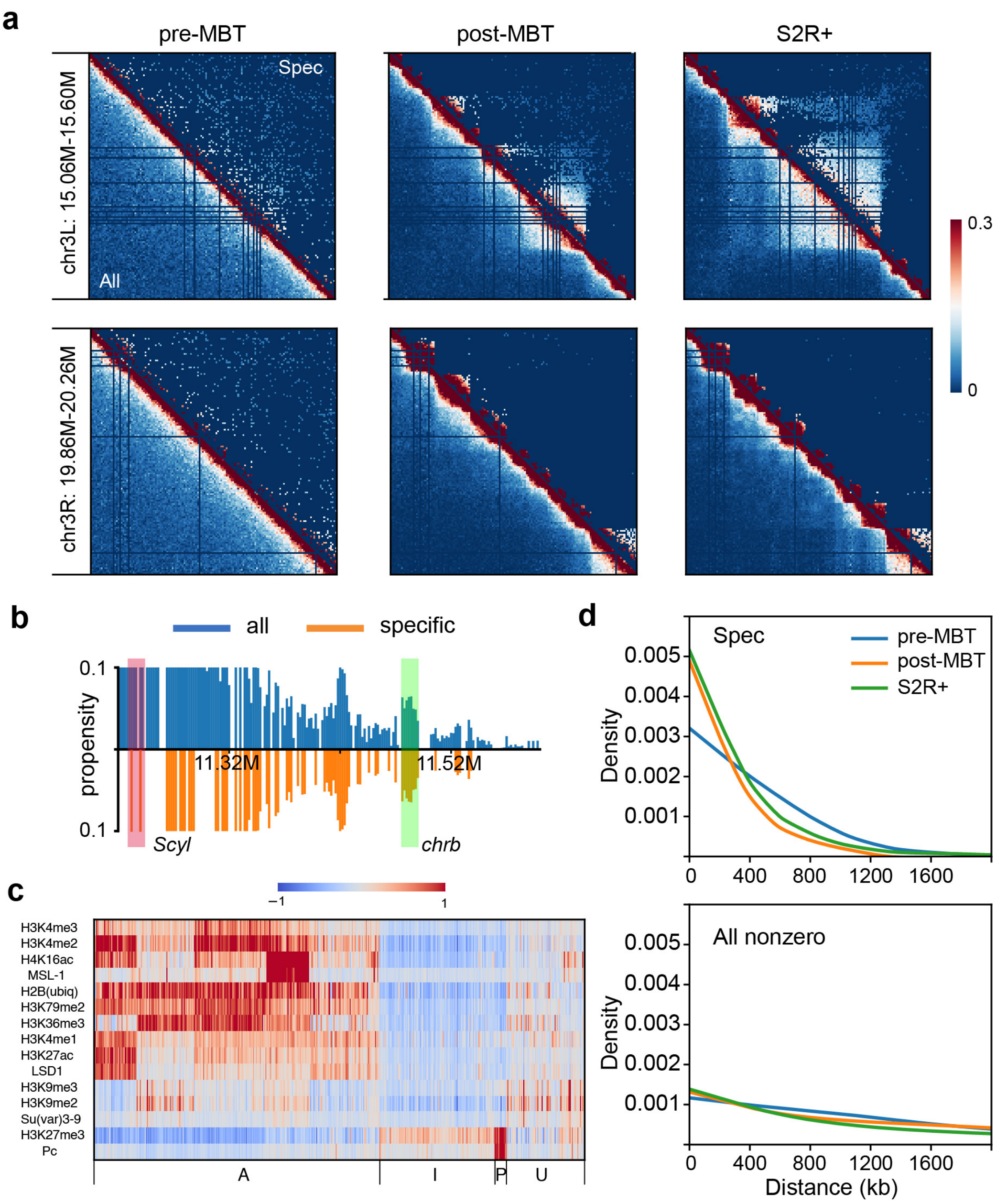
a
pre-MBT
post-MBT
S2R+
Spec
All
chr3L: 15.06M-15.60M
chr3R: 19.86M-20.26M
0.3
0
b
all
specific
propensity
0.1
0.1
11.32M
11.52M
Scyl
chrb
c
−1
1
H3K4me3
H3K4me2
H4K16ac
MSL-1
H2B(ubiq)
H3K79me2
H3K36me3
H3K4me1
H3K27ac
LSD1
H3K9me3
H3K9me2
Su(var)3-9
H3K27me3
Pc
A
I
P
U
d
Spec
pre-MBT
post-MBT
S2R+
Density
0.005
0.004
0.003
0.002
0.001
0
400
800
1200
1600
All nonzero
Distance (kb)

Supplementary Figure 3. Overview of specific interactions. **(a)** Specific interactions identified from an inactive region (top) and an active region (bottom) in three cell types at different developmental stages. Lower-left triangle represents all Hi-C interactions, upper-right triangle represents the specific interactions we identified. Cell types from left to right are embryos at cycles 9–13, embryos at stages 5–8 and S2R+ respectively. **(b)** A virtual 4C plot shows the specific interactions identified in a 380 kb region of S2R+. Red bar represents the anchor which contains the gene *Scyl*, green bar represents a specific interaction that targets the gene *chrb*. Note that bins immediately neighboring the bin containing *chrb* also exhibit elevated interaction propensity, in contrast to that of *Bsg25A/slam* shown in Figure 2b. This is likely related to the much longer genomic distance of $\geq$ 1.6 Mb between *Bsg25A* and *slam*, compared to $\sim$ 250 kb between *Scyl* and *chrb* shown here. As both Figure 2b and Supplementary Figure 3b depict propensities rather than absolute frequencies, it is not apparent that the elevated propensity of *Bsg25A-slam* is based on read-outs of much smaller absolute frequencies prior to normalization (8) compared to that of *Scyl-chrb* (37). Due to this longer genomic distance, there is overall lowered absolute frequencies of ligation between bins neighboring *slam* and the bin of *Bsg25A* and its neighbors in Hi-C experiment. Since we use a rather aggressively stringent criterion in our null model (FDR $\leq$ 0.01), such interactions of lower frequency with bins neighboring *slam* are not selected, in contrast to the strong elevated signal of interaction we do detect at the bin containing *slam* and its neighbors.Because of its 3D geometric basis, our method is likely more accurate in accounting for the bias due to genomic distance (1.6 Mb vs 250 kb), and therefore potentially more powerful in detecting specific long range interactions. This is evidenced from results of comparing specific interactions by our method and those by the Fit-Hi-C [14] and GOTHiC [15], where our method detects more long-range ($\geq$ 500 kb) interactions (26.2% vs. 17.3% and 1.35% for Fit-Hi-C and GOTHiC, respectively). **(c)** Hierarchical clustering of the genomic regions based on the ChIP-chip signals of 15 different histone modifications or non-histone proteins. All regions are categorized into 4 types, they are active(A), inactive(I), polycomb-repressed(P) and undetermined(U). The last type is not included in the following analysis. **(d)** Density curves of the specific interactions (top) and all non-zero Hi-C interactions (bottom) in three cell types.

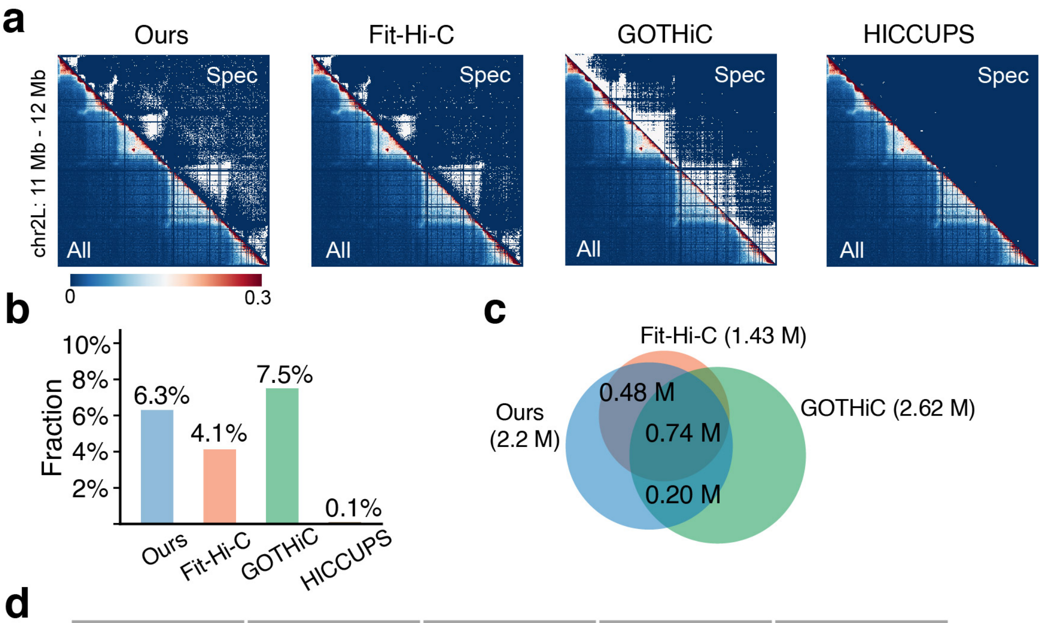

d

Methods	Ours	Fit-Hi-C	GOTHiC	HICCUPS
r	0.949	0.915	0.941	0.548
r'	0.666	0.636	0.624	0.323

Supplementary Figure 4. Comparison of specific interactions among different methods. **(a)** Distributions of all and specific interactions by our method, Fit-Hi-C, GOTHiC and HICCUPs in a 1 Mb region (chr2L: 11–12 Mb). Lower left triangle shows all Hi-C contact frequencies, upper right triangle shows specific interactions (white and dark blue colors represent background and specific interactions). **(b)** Fractions of genome-wide specific interactions within 4 Mb genomic distance using different methods. **(c)** Overlap of specific interactions among our method, Fit-Hi-C and GOTHiC. **(d)** Pearson correlation coefficient r and distance-adjusted correlation coefficient r' between Hi-C and simulated contact frequencies obtained from polymer ensembles constructed using specific interactions of our method, Fit-Hi-C, GOTHiC and HICCUPS.

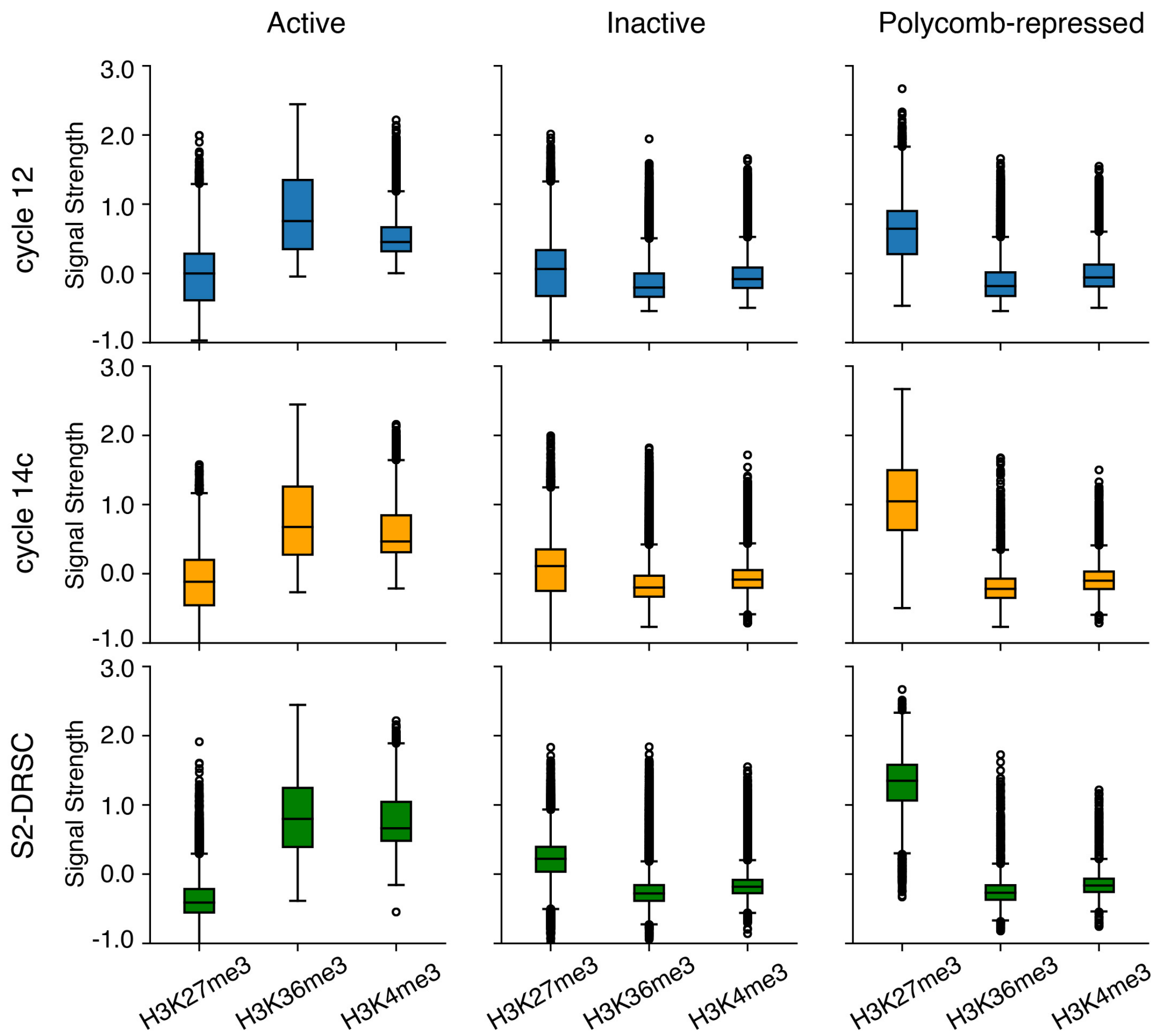

Supplementary Figure 5. Distributions of H3K27me3, H3K36me3 and H3K4me3 ChIP-seq or ChIP-chip quantile-normalized signals from embryos at cycle 12, cycle 14c, and S2-DRSC. The three chromatin states (Active, Inactive and Polycomb-repressed) are assigned based on S2 markers. ChIP-seq and ChIP-chip data are taken from the GEO database (H3K27me3, H3K4me3 and H3K36me3 of cycle 12 are from GSM1424916, GSM1424909 and GSM1424919, H3K27me3, H3K4me3 and H3K36me3 of cycle 14c are from GSM1424918, GSM1424911 and GSM1424921). Processed wiggle files downloaded from the database are quantile-normalized before comparison. The resolution is 2 kb.

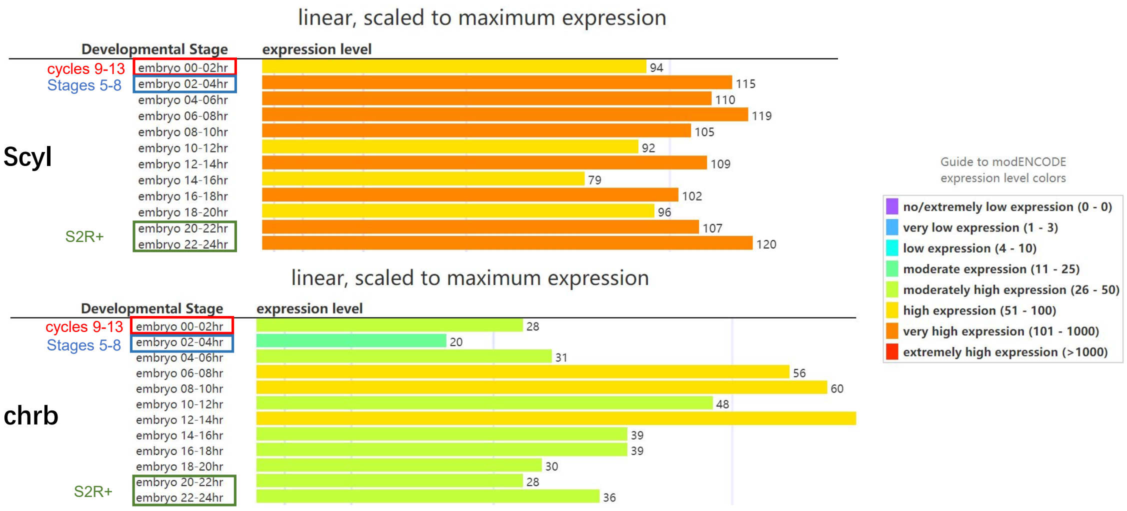

Supplementary Figure 6. Expression level of gene *Scyl* (top) and *chrb* (bottom) during *Drosophila* embryogenesis (data from Flybase (`https://flybase.org/`)). Both genes are expressed at all developmental stages, at least moderately. Therefore, the frequent interactions observed in our model are unlikely due to Polycomb repression. *Scyl* has higher expression level at both stages 5–8 (blue box) and S2R+ (green box). This is overall consistent with the higher contact frequency of *Scyl*/enhancer interaction in later embryos (Supplementary Figure 9g). *chrb* has a low expression level at stages 5–8 but its expression is elevated in S2R+. This is consistent with the enhanced *chrb*/enhancer contact frequency in S2R+ (Supplementary Figure 9h). A full mechanistic understanding of Scyl/chrb interaction and how these two genes negotiate with the enhancer may require considerations of other possible mediators (such as architectural proteins) participating in the regulation process. These, however, are not explicitly modeled in this study.

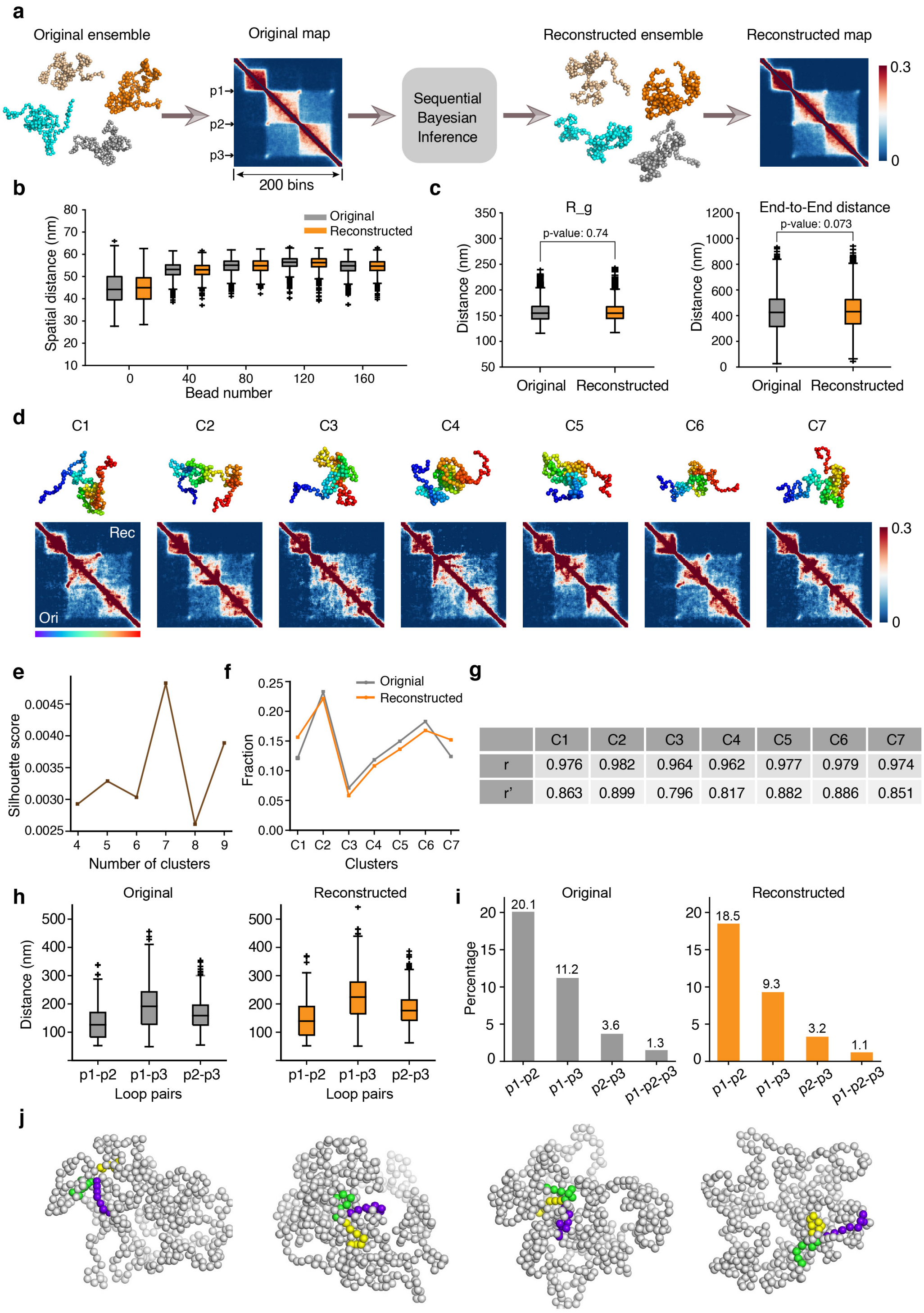

	C1	C2	C3	C4	C5	C6	C7
r	0.976	0.982	0.964	0.962	0.977	0.979	0.974
r'	0.863	0.899	0.796	0.817	0.882	0.886	0.851

Supplementary Figure 7. An illustration of in-silico simulation procedure for model verification. **(a)** First, we construct an original chromatin polymer ensemble consisting of 50,000 chains and aggregate the single-chain contact maps into a simulated Hi-C contact map; we then apply our sequential Bayesian inference framework and reconstruct another polymer ensemble consisting of 50,000 chains solely from the simulated Hi-C contact map. **(b)** Distance distributions of 5 specific nodes (0-th, 40-th, 80-th, 120-th and 160-th) with all other nodes (less than 80 nm threshold). **(c)** Distributions of radius of gyration (R_g) and End-to-End distance of the original and the reconstructed ensemble. Wilcoxon rank sum test is used for *p*-value calculation. **(d)** Hierarchical clustering of chromatin polymer chains after combining the original and reconstructed ensemble (randomly select 5,000 chains from each ensemble for clustering). **(e)** Silhouette scores for different choices of cluster numbers. **(f)** Distributions of cluster proportions of the original and the reconstructed ensemble. **(g)** Pearson correlation coefficient r and distance-adjusted correlation coefficient r' between aggregated contact maps from the original and the reconstructed ensemble for each cluster. **(h)** Distance distributions of p1-p2, p1-p3, and p2-p3 looping contact pairs labeled in **(a)** for the original and reconstructed ensembles. **(i)** Percentages of p1-p2, p1-p3, p2-p3 looping pairs and p1-p2-p3 three-body contact pairs in the original and reconstructed ensembles. **(j)** A toy ensemble of four chromatin polymer chains generated under the $a\ priori$ condition that the three loci p1, p2, and p3 interact solely as a triplet p1-p2-p3. The resulting ensemble is generated using knock-in perturbations of the CHROMATIX method [17], with loci p1, p2, and p3 shown as green, yellow, and purple beads, respectively.

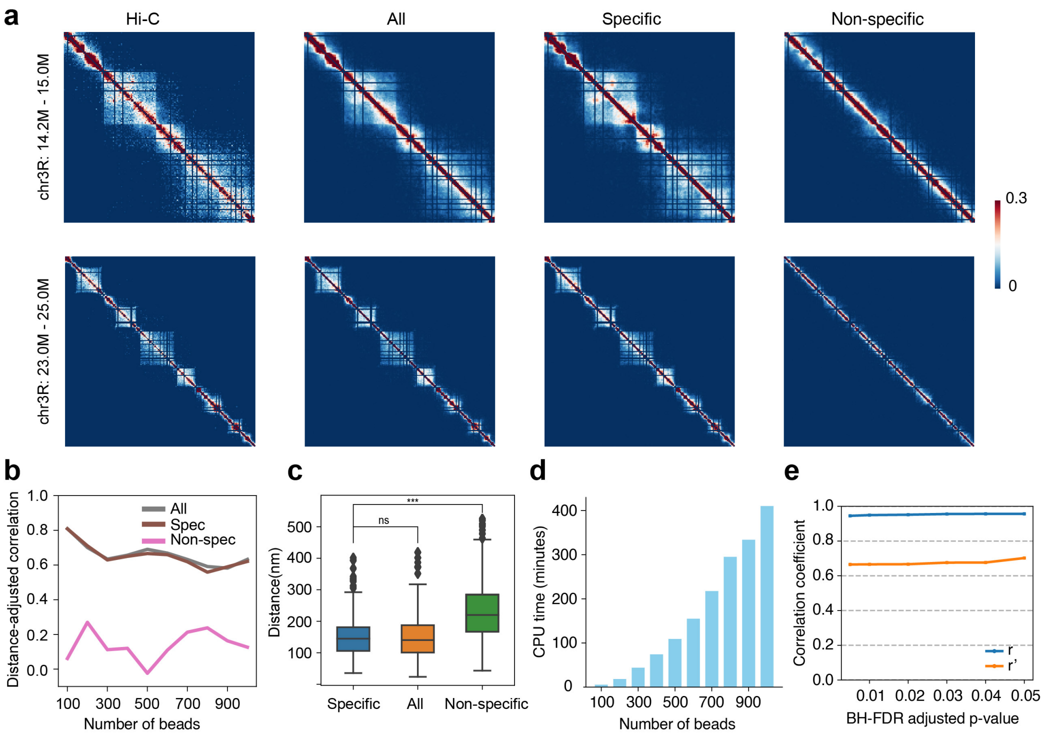

Supplementary Figure 8. Specific interactions are sufficient to drive chromatin folding. **(a)** Simulation results of 2 regions of different genomic lengths. Heat maps from left to right represent Hi-C propensities, simulated contact probabilities using specific, all and non-specific interactions respectively. Resolution is 2 kb. **(b)** Distance-adjusted correlation coefficient of the simulated contact probabilities and Hi-C propensities in ten regions of different genomic lengths. The number of beads ranges from 100 to 1000. **(c)** Distance distributions of a control interaction of the same length as the loop in Figure 3D. The two contacting loci correspond to bead No. 215 and bead No. 235. *** represents two-sided Wilcoxon rank sum test p-value $\leq$ 0.001, ns means p-value $>$ 0.05. **(d)** averaged CPU time (minutes) for constructing 3D polymer chains (50,000 chains, 20 Intel(R) Xeon(R) E5-2650 CPU cores) using frequencies of specific interactions for the ten regions. **(e)** Pearson correlation coefficient (r) and distance-adjusted correlation coefficient (r') between Hi-C and simulated contact maps under different thresholds of BH-FDR adjusted p-value for the 1 Mb region of chr2L: 11.0–12.0 Mb.

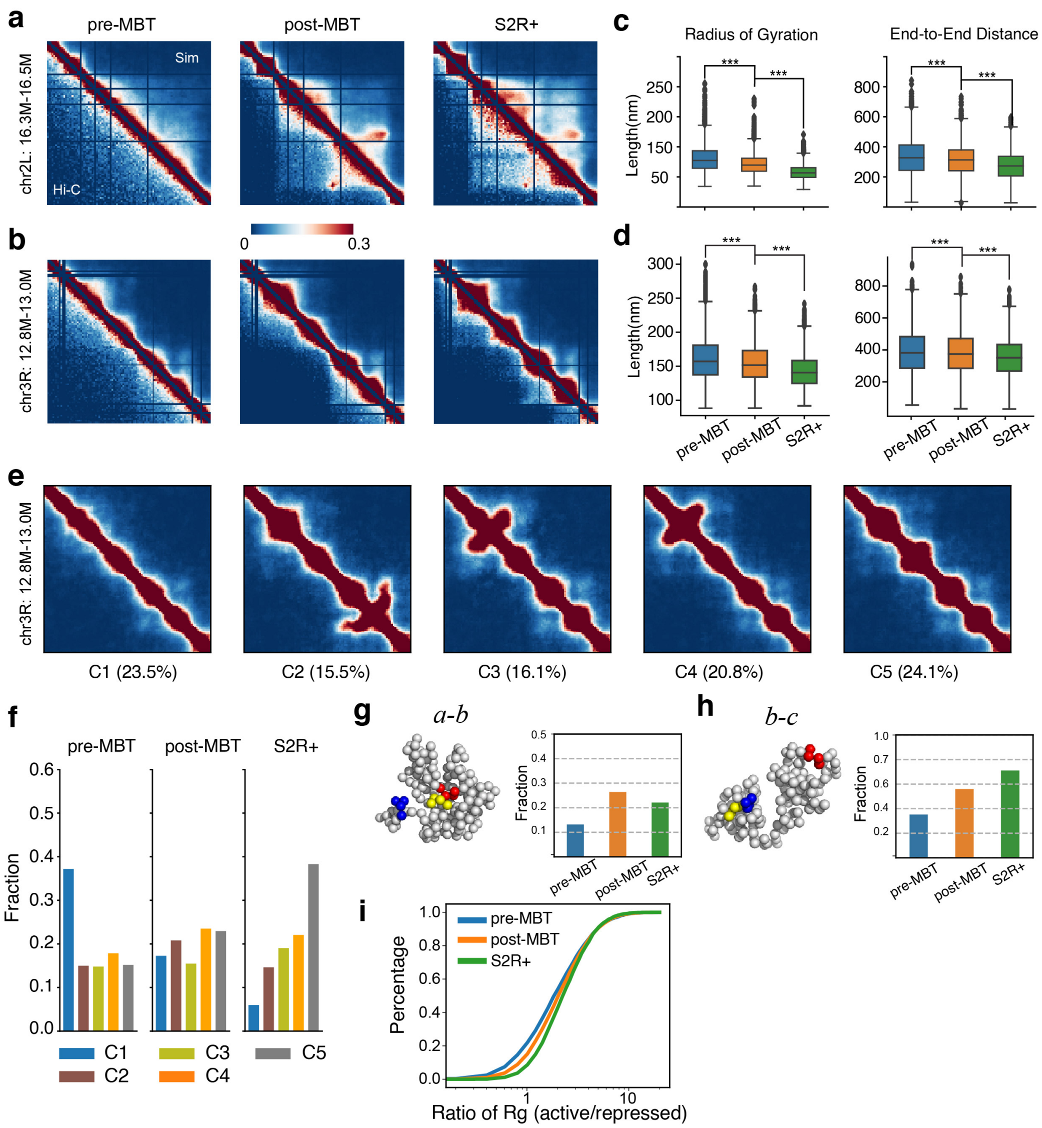
a
pre-MBT
post-MBT
S2R+
chr2L: 16.3M-16.5M
Sim
Hi-C
b
chr3R: 12.8M-13.0M
0
0.3
c
Radius of Gyration
End-to-End Distance
Length(nm)
d
pre-MBT
post-MBT
S2R+
e
chr3R: 12.8M-13.0M
C1 (23.5%)
C2 (15.5%)
C3 (16.1%)
C4 (20.8%)
C5 (24.1%)
f
Fraction
C1
C2
C3
C4
C5
g
a-b
h
b-c
i
Percentage
Ratio of Rg (active/repressed)

Supplementary Figure 9. Heterogeneity in modeled single-cell chromatin conformations, chromatin compaction, and dynamic changes of a three-body interaction unit during *Drosophila* embryogenesis. Simulation results of a polycomb-repressed region**(a)** and an active region**(b)** in three cell types at different developmental stages. Lower-left triangles represent the Hi-C propensities, upper-right triangles represent the simulated contact probabilities. Cell types from left to right are embryos at cycles 9–13, at stages 5–8 and S2R+ respectively. **(c)** Distributions of Radius of gyration (left) and end-to-end distance (right) of the region from **(a)**, *** represents two-sided Wilcoxon rank sum test p-value < 0.001. **(d)** Distributions of Radius of gyration (left) and End-to-End distance (right) of the region from **(b)**. *** represents two-sided Wilcoxon rank sum test p-value < 0.001. **(e)** Hierarchical clustering of 3D chromatin configurations of all three cell types in the active region of chr3R: 12.8–13.9 Mb. Proportions of the 5 clusters are labeled below the aggregated heat maps. **(f)** Proportions of the 5 clusters in each cell type. **(g)** Left figure shows an example of a two-body interaction between the promoter of gene *Scyl* and the putative enhancer which is labeled in Figure 4e. Right barplot shows the fractions of this two-body interaction in three different cell types. **(h)** Left figure shows an example of a two-body interaction between the promoter of gene *chrb* and the putative enhancer which is labeled in Figure 4e. Right barplot shows the fractions of this two-body interaction in three different cell types. **(i)** Cumulative percentage curves of ratios of Radius of Gyration between chromatin chains of the active region shown in **(b)** to chromatin chains of the repressed region shown in **(a)**.

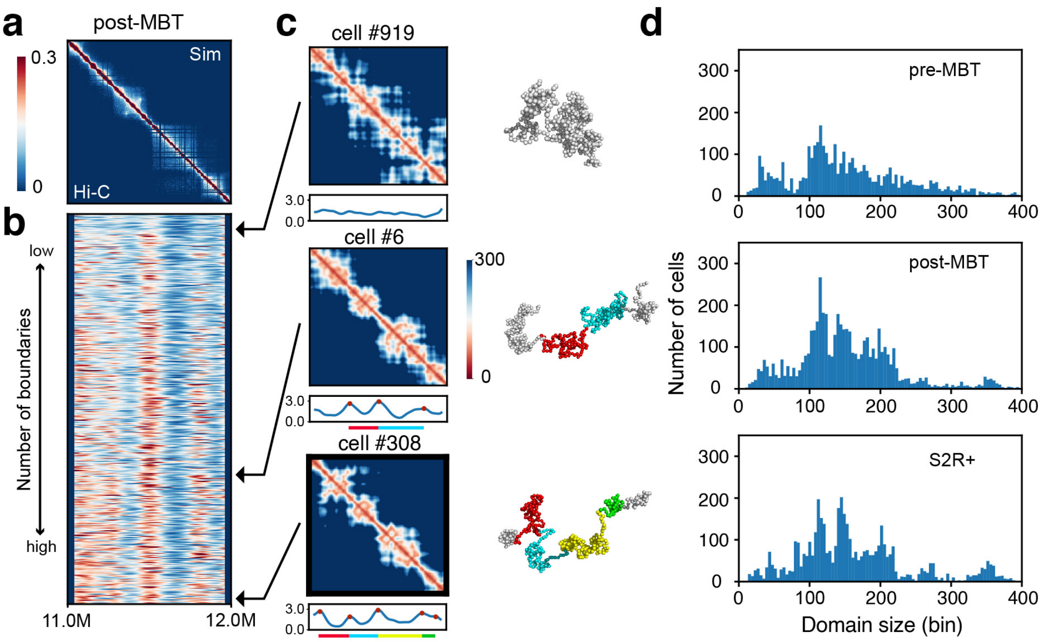

Supplementary Figure 10. TAD-like structures in modeled single cells during *Drosophila* embryogenesis. **(a)** Simulation results of the region (chr2L: 11.0–12.0 Mb) in embryos at stages 5-8. Lower-left triangle represents the Hi-C propensities, upper-right triangle represents the simulated contact probabilities. The resolution is 2 kb. **(b)** Boundary strength profiles of 5,000 configurations in embryos at stages 5-8. The order is based on the number of domain boundaries. **(c)** Three representative single-cell spatial-distance maps(on the left) and corresponding visualizations(on the right) in embryos at stages 5-8 with different boundary numbers, the number of boundaries from top to bottom is 0, 3 and 5 respectively. The arrows indicate their positions in the boundary strength profiles on the left. Boundary strength curves are drawn under the spatial-distance maps, with red dots representing the local maxima identified as domain boundaries. Bars in different colors under the boundary strength curves represent different domains identified in that configuration, which are also labeled in the 3D visualizations. **(d)** Distributions of domain sizes in three cell types. Domains are identified in the region shown in **(a)**.

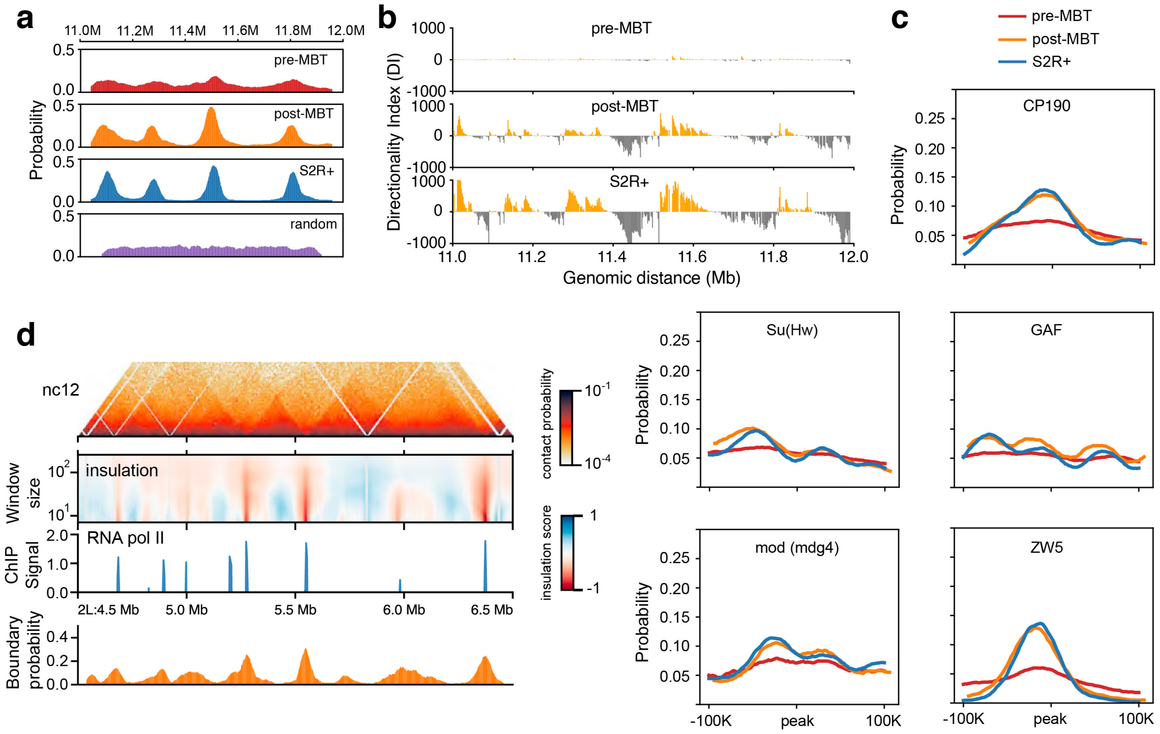

Supplementary Figure 11. Simulated single-cell domain boundaries among cells at three developmental stages of embryogenesis (pre-MBT (cycles 9–13), post-MBT (stages 5–8) and S2R+ derived from late embryos). **(a)** Distributions of domain boundary probabilities along genomic positions of the region shown in Figure 5A (chr2L: 11.0–12.0 Mb) in the three cell types and the random polymer ensemble. All ensembles consists of 5,000 configurations. The diminished random probabilities at the start and end of the region are boundary effects and can be corrected. **(b)** Directionality Index (DI) for cells at the three different stages in the region of chr2L: 11.0 – 12.0 Mb shown in Figure 5a. **(c)** Enrichment curves of the averaged domain boundary probabilities at the binding peaks of 5 different insulator proteins. These 5 insulator proteins are CP190, GAF, ZW5, Su(Hw) and mod(mdg4). **(d)** Preference of simulated single-cell domain boundaries at pre-MBT boundary positions in Hi-C data of Hug et al. The heat map, insulation and RNA pol II tracks are from Hug et al [29] (with permission). The bottom track shows the probabilities of simulated single-cell domain boundaries in the same region of chr2L: 4.5 – 6.5 Mb.

Supplementary Tables

Supplementary Table 1. Pearson Correlation and distance-adjusted correlation coefficients of the simulated contact probabilities using specific interactions and the Hi-C propensities in ten regions with different genomic lengths.

Region	Number of beads	Specific r	Specific r'	All r	All r'	Non-specific r	Non-specific r'
chr2L: 16,310,000-16,510,000	100	0.981	0.809	0.984	0.810	0.536	0.061
chrX: 15,960,000-16,360,000	200	0.984	0.711	0.987	0.701	0.578	0.269
chr3R: 12,300,000-12,900,000	300	0.955	0.629	0.967	0.634	0.540	0.112
chr3R: 2,200,000-3,000,000	400	0.955	0.650	0.965	0.657	0.584	0.120
chr2L: 11,000,000-12,000,000	500	0.949	0.666	0.949	0.691	0.533	-0.024
chr2L: 14,200,000-15,400,000	600	0.943	0.660	0.942	0.669	0.559	0.109
chr3R: 1,600,000-3,000,000	700	0.943	0.619	0.953	0.632	0.580	0.213
chr2L: 16,800,000-18,400,000	800	0.906	0.559	0.914	0.591	0.492	0.238
chrX: 1,700,000-3,500,000	900	0.980	0.590	0.981	0.582	0.537	0.163
chr3R: 23,000,000-25,000,000	1000	0.946	0.621	0.955	0.632	0.514	0.126

Supplementary Table 2. modENCODE datasets for Hierarchical clustering of the specific interactions in S2R+.

Marker	modENCODE ID
H3K4me3	305
H3K4me2	965
H4K16ac	319
MSL-1	3293
H2B(ubiq)	290
H3K79me2	307
H3K36me3	303
H3K4me1	304
H3K27ac	296
H3K27me3	298
LSD1	3949
H3K9me3	313
H3K9me2	311
Su(var)3-9	2673
Pc	326

Supplementary Methods

Null model construction via Fractal Monte Carlo

We apply a novel Fractal Monte Carlo approach(under review) derived from C-SAC [13] to construct our physical null model. This approach is based on the sequential importance sampling (SIS) [8, 17]. We model chromatin fibers as random polymer chains consisting of self-avoiding beads within a defined space. For each random polymer chain $X_n^{(k)}$, we place the beads one by one until it reaches the wishful polymer length of n.

We generate the intermediate sample $X_t^{(k)}$ given that we've already produced $X_{t-1}^{(k)}$. $X_t^{(k)}$ is generated according to the intermediate sampling distribution,

$$\begin{aligned} X_t^{(k)} &\sim P_s\left(X_t^{(k)}|X_{t-1}^{(k)}\right) \\ &= P_s\left(x_1^{(k)}, x_2^{(k)}, \cdots, x_t^{(k)}|x_1^{(k)}, x_2^{(k)}, \cdots, x_{t-1}^{(k)}\right) \end{aligned} \qquad \text{(Supplementary Equation 1)}$$

We assume the null model is a uniform-energy system, thus the intermediate sampling follows a uniform distribution

$$P_s\left(X_t^{(k)}|X_{t-1}^{(k)}\right) = \frac{1}{N_t^{(k)}} \quad , \qquad \text{(Supplementary Equation 2)}$$

where $N_t^{(k)}$ is the number of all possible valid positions of $x_t^{(k)}$ given positions of the previously placed beads $x_1^{(k)}, x_2^{(k)}, \cdots, x_{t-1}^{(k)}$.

Our target distribution $P_t^{(k)}$ is a constant, which is reversely proportional to the number of all potential valid polymer chains of length n that satisfy the physical constraints(self-avoiding property and nuclear confinement). According to the theory

of SIS, the importance weight $w^{(k)}$ of the k-*th* polymer chain is defined as

$$\begin{aligned}
w^{(k)} &= \frac{P_t\left(X_n^{(k)}\right)}{P_s\left(X_n^{(k)}\right)} \\
&\propto \frac{1}{P_s\left(X_1^{(k)}\right) P_s\left(X_2^{(k)}|X_1^{(k)}\right), \cdots, P_s\left(X_t^{(k)}|X_{t-1}^{(k)}\right)} \\
&\propto N_1^{(k)} N_2^{(k)}, \cdots, N_n^{(k)}
\end{aligned}$$

(Supplementary Equation 3)

For polymer simulations with large number of beads, the final weight $w^{(k)}$ would be very low. To solve this problem, we decide to apply the resampling procedure [8] to improve the importance sampling weights of our random polymers. The procedure is as follows:

(1) At the m step, giving the intermediate ensemble of the polymers $X_m^{(1)}$, $X_m^{(2)}$, $\cdots$, $X_m^{(N)}$, we sample N numer of new polymers with replacement from the existing ensemble. Each original sample $X_m^{(k)}$ will be selected with the resampling probability $P_{rs}\left(X_m^{(k)}\right)$, which equals to

$$P_{rs}\left(X_m^{(k)}\right) = \frac{w_m^{(k)}}{\sum_{k=1}^{N} w_m^{(k)}} \qquad \text{(Supplementary Equation 4)}$$

(2) Due to the resampling process, the final polymer chains are actually generated from a modified distribution $P_s^*(\cdot)$. To eliminate the resampling bias, we multiply the importance weight of each selected sample by a factor $\frac{1}{P_{rs}(.)}$. The corrected weight $w_m^{*(k)}$ would be

$$w_m^{*(k)} = \frac{w_m^{(k)}}{P_{rs}\left(X_m^{(k)}\right)} \qquad \text{(Supplementary Equation 5)}$$

To largely explore the sampling space and increase the sampling diversity, our fractal Monte Carlo approach consists of two layers of sequential importance sampling processes. The top layer is the parent sampling where several landmarks are labeled.

Each parent sample grows to the landmark lengths by deferring to the parallel child samplings. Each child sampling is a SIS process, in which a resampling quality-control procedure is performed. At each landmark, the parent will pick only one polymer chain from the child ensemble based on the their importance weights. This growing process will continue until the parent samples reach their wishful lengths. By this two-layer Fractal Monte Carlo approach, we achieve deep sampling of random polymer chains efficiently with a rich sampling diversity.

To evaluate the quality of the random polymer chains, we calculate the effective sample size (ESS) [8] which is defined as

$$ESS = \frac{(E(W))^2}{(E(W))^2 + Var(W)} \quad , \qquad \text{(Supplementary Equation 6)}$$

where W represents the importance weights of the ensemble $w^{(1)}, w^{(2)}, \cdots, w^{(N)}$. We find the ESS of the polymer ensemble is generally very low(less than 0.01). By examining the weight of each polymer chain, we find a few of samples have extremely high weights that dominate the whole ensemble. We decide to remove these outliers based on a 1.5*IQR threshold [10], all samples with importance weights between $Q1 - 1.5 * IQR$ and $Q3 + 1.5 * IQR$ are selected to constitute our final ensemble, here Q1 and Q3 are the 25-*th* and 75-*th* percentiles of the ensemble weights respectively.

Our null model is based on the considerations that the excluded volume of self-avoiding chromatin chains and the confinement of the cell nucleus impose strong restrictions on the available space for chromatin. As experimental treatments can cross-link genomic elements within certain spatial distances, regardless of whether specific interactions exist, many Hi-C detected frequencies may be due to such random non-specific bystander collision contacts [21]. Our null model is designed to identify Hi-C contact pairs which cannot be accounted for by such random collision due to the physical properties of excluded volume, chain connectivity, and volume confinement. The specific contact pairs are selected rather conservatively following a stringent *p*-value and FDR criterion.

Previous studies showed that this random polymer model of self-avoiding con-

nected polymers with excluded volume can reproduce experimentally observed scaling relationships of contour-length vs genomic distance (as measured by FISH) and looping probability vs genomic distance (as measured by Hi-C) [13]. With a few additional biological landmarks, it has also been successful in reproducing genome-wide Hi-C contact maps of budding yeast [20].

An intuitive data-driven approach for identifying specific Hi-C interactions is to take the average along each off-diagonal and then consider specific contacts to be those that have abnormally high (or low) frequencies relative to their same-distance peers. This data-driven approach has some disadvantages. First, it uses the measured Hi-C to estimate a background model serving as a null distribution. This can bias the calculation of statistical significance of Hi-C contacts, as the specific contacts to be detected themselves are integral to the generation of the null distribution. Second, as there are less and less contact pairs for longer-range off-diagonals, detecting specific long range contact pairs becomes difficult without resorting to certain *ad-hoc* interpolation/extrapolation across neighboring diagonals. Our method avoids this difficulty, as it has a completely decoupled random model, which can generate a sufficiently large number of samples, allowing reliable detection of long-range interactions.

Bootstrap the polymer ensemble via Bag of Little Bootstrap (BLB)

We estimate the statistical significance of each Hi-C interaction by bootstrapping the random polymer chains through a strategy called Bag of Little Bootstrap (BLB) [7]. It includes the following steps:

(1) Given the size α of each BLB subset, we sample α new polymers without replacement from the original polymer ensemble. We pick α based on

$$\alpha = n^{\beta} \quad , \qquad \text{(Supplementary Equation 7)}$$

where n is the size of the original ensemble, β is a scaling parameter. When $\beta = 1$, this BLB procedure is equivalent to the canonical bootstrap, here we have $\beta = 0.6$.

(2) After we get the BLB subset, we generate the BLB ensemble by sampling n new polymers with replacement from the BLB subset generated in the last step.

(3) We repeat the last step r times and compute the contact probabilities of the BLB ensemble by averaging all the repeated results, here we have $r = 50$.

We generate 5,000 BLB random ensembles for each null model construction. Given the large size of our original ensemble, it's time-consuming to apply the canonical bootstrap which samples the new polymers to the original ensemble size n. Through this BLB strategy, we could obtain the bootstrap ensembles in a much more efficient way.

Identification of the genome-wide specific interactions

We use a sliding window which has the same size of the random polymers to scan across each chromosome with a step of 1 bin. For each step, we assign a p-value to every Hi-C interaction in that current sliding window based on the proportion of the random contact probabilities computed from the BLB random ensembles that exceed the relative Hi-C propensity. After the sliding process, the majority of the Hi-C interactions have multiple p-values due to the overlap of the regions in the sliding window at different steps, we take the average of all the p-values that belong to the same Hi-C interaction. After the p-value calculation, we use the BH-FDR [1] method to correct all the statistical p-values, Hi-C interactions with FDR-adjusted p-values below a threshold of 0.01 are selected to be our specific interactions.

We apply the same procedure to five chromosomes of *Drosophila* (chr2L, chr2R, chr3L, chr3R and chrX) with a sliding window size of 4 Mb, thus we could identify all the specific interactions with a separating genomic distance less than 4 Mb across the entire *Drosophila* genome.

Comparison of specific interactions among different methods

We compared specific interactions identified using our method with those from Fit-Hi-C [14] (v 2.0.7), GOTHiC [15] (v 1.22.0), and HICCUPS [16] (v 1.14.08). For

Fit-Hi-C, we use parameters "`-r 2000 -p 2`". A bias file generated by ICE is also used for the `-t` option. Fit-Hi-C-specific interactions are selected based on the criteria of q-value ≤ 0.01. For GOTHiC, we rearrange our valid Hi-C ligation products to HiCUP output format as required by GOTHiChicup, after removal of PCR duplicates, self-ligation and dangling reads. We perform GOTHiC calculations via GOTHiChicup using default parameters. Hi-C contacts with q-value ≤ 0.01 are identified as GOTHiC-specific interactions. For HICCUPS, we take the implementation from the juicer-tools (`https://github.com/aidenlab/juicer`) with options

"`--cpu -m 1024 -k KR -r 2000 -p 4 -i 10 -d 10000 -f 0.01 --ignore_sparsity`".

Enriched pixels with FDR ≤ 0.01 are then selected. We constructed a separate 3D chromatin ensemble for each method using the respective specific interaction frequencies; all 3D ensembles were generated via sequential Bayesian inference framework. Each ensemble consisted of 50,000 single-chain chromatin polymers.

The specific interactions for a 1 Mb region (chr2L: 11–12 Mb) are shown in Figure S4a. Overall, there are 6.3% (ours), 4.1% (Fit-Hi-C), 7.5% (GOTHiC) and 0.1% (HICCUPS) of genome-wide S2R+ Hi-C contacts within 4 Mb genomic distance that are identified to be specific, respectively (Supplementary Figure 4b). Despite a lower fraction retained than GOTHiC (6.3% vs. 7.5%), our method identifies more long-range specific interactions (26.2% vs. 1.35% with separating distance ≥ 500 kb, respectively). The number of specific interactions in 10^6 (M) for the whole genome is shown in the Venn diagram of Supplementary Figure 4c, along with the numbers of the intersections. Specifically, 85.2% and 36.0% of Fit-Hi-C and GOTHiC specific interactions are also identified by our method.

We further used our folding algorithm and constructed 3D ensembles of chromatin structures using contact frequencies of specific interactions obtained with these methods for the 1 Mb region of chr2L:11–12 Mb. We evaluate similarities between simulated and Hi-C contact frequencies based on Pearson correlation coefficient r and distance-adjusted correlation coefficient r. While our folding algorithm can generate 3D chromatin ensembles using specific interactions identified by our null model, our

folding algorithm can also utilize Fit-Hi-C and GOTHiC specific interactions which are also sufficient for generating 3D ensembles. However, results using our null model have the highest r and r'-values, and are most similar to the original Hi-C measurements (Supplementary Figure 4d).

Hierarchical clustering of the specific interactions

We download 15 ChIP-chip datasets of different histone modification and protein markers from the modENCODE database (Supplementary Table 2). These 15 markers indicate different biological functions [6], from active promoter (H3K4me2, H3K4me3, H2B (ubiq), H3K79me2) and active enhancer (H3K4me1, H3K27ac and LSD1) to heterochromatin (H3K9me3, H3K9me2 and Su(var)3-9) and polycomb-repressed region (H3K27me3 and Pc). H4K16ac and MSL-1 are associated with active regions that primarily at chromosome X, H3K36me3 is a marker that indicates the transcription elongation.

We calculate the averaged signals for each genomic region of a bin size. We use a Hierarchical clustering program (Agglomerative Clustering) from the scikit-learn package (v 0.21.2) [9] to categorize all genomic regions into 8 different clusters based on the averaged signals of these 15 markers in each region. These 8 clusters represent 4 different chromatin states [6], they are active (A), inactive (I), polycomb-repressed (P) and Undetermined (U) respectively. The last cluster (U) is not included in the following analysis. The number of clusters is based on the Silhouette scores from a testing subset which contains 1/10 genomic regions that are randomly picked.

After the classification of each genomic region, we categorize all specific interactions into 6 interaction clusters based on the chromatin states of the two contacting regions of each specific interaction. These 6 clusters are active-active (A-A), active-inactive (A-I), active-polycomb (A-P), inactive-inactive (I-I), inactive-polycomb (I-P) and polycomb-polycomb (P-P) respectively. To assess possible changes in the distribution of different interaction types, we use the 15 markers of S2 to categorize all specific interactions from each cell type, as the chromatin classification based on S2 cells is robust across embryos at cycle 12 and cycle 14c. This is supported by

an analysis following the approach of [18] to examine the three representative histone modifications (H3K37me3, H3K36me3 and H3K4me3). Supplementary Figure 5 shows that after signals of histone modifications in 2 kb bins are aggregated over the same three chromatin states (Active, Inactive and Polycomb-repressed) defined by S2 markers, their distributions are unchanged in embryos at cycle 12 (during cycles 9–13) and cycle 14c (during stages 5–8). We further calculate the proportions of interaction clusters of each cell type in the following analysis.

Deep sampling chromatin structures via implementing a sequential Bayesian inference framework

The Bayesian inference framework has been successfully applied to generate chromatin structures of yeast [12]. However, in the previous framework, they assume there is a simple reverse relationship between the Hi-C contact frequencies and the spatial distances, each polymer chain is constructed under the same assumption that the spatial distance of a certain pair of beads should be a spurious value that computed according to a reverse function. Considering the reality of the Hi-C experiments, we construct chromatin structures based on a more reasonable assumption that only regions which are in close spatial proximity can form Hi-C ligation products. Besides, we construct the polymer chains sequentially, which overcomes the problem that it's much harder to find the feasible configuration consistent with Hi-C through the optimization process after placing all the beads at once in the initial ensemble.

Our sequential Bayesian inference framework is shown in Supplementary Figure 1. Specifically, we construct the polymer ensemble simultaneously by optimizing the probability $P\left(E|H\right)$ according to the Bayes' function

$$P\left(E|H\right) = \frac{P\left(H|E\right)P\left(E\right)}{P\left(H\right)} \quad , \qquad \text{(Supplementary Equation 8)}$$

where E is the polymer ensemble consists of $X^{(1)}, X^{(2)}, \cdots, X^{(N)}$, H represents the constraints of the selected Hi-C interactions. We generate the polymers in a sequential

way, thus,

$$\begin{aligned} P(E|H) &= P(E_1|H_1)\,P(E_2|H_2)\cdots P(E_n|H_n) \\ &= \prod_{t=1}^{n} P(E_t|H_t) \\ &\propto \prod_{t=1}^{n} P(H_t|E_t)\,P(E_t) \quad , \end{aligned} \qquad \text{(Supplementary Equation 9)}$$

where H_t represents the constraints of the Hi-C propensities $p_{obs}(x_1, x_t)$,$p_{obs}(x_2, x_t)$,$\cdots$, $p_{obs}(x_{t-1}, x_t)$. E_t is the intermediate polymer ensemble consists of $X_t^{(1)}, X_t^{(2)}, \cdots, X_t^{(N)}$ at the t step. $P(E|H)$ contains two terms:

(1) $P(H_t|E_t)$ is the term that evaluates the similarities between the simulated contact probabilities of the intermediate polymer ensemble E_t and the Hi-C propensities H_t. We model this term through a Poisson distribution, which could eliminate the biases introduced by the dominance of large contact counts [11]. According to the Poisson distribution function, $P(H_t|E_t)$ could be represented as

$$P(H_t|E_t) = \prod_{m=1}^{t-1} \frac{\lambda_{mt}^{C_{sim}(x_m,\ x_t)}}{C_{sim}(x_m,\ x_t)!} \exp(-\lambda_{mt}) \quad , \qquad \text{(Supplementary Equation 10)}$$

where $\lambda_{mt} = C_{obs}(x_m,\ x_t)$, which is equivalent to the product of the Hi-C propensity $P_{obs}(x_m,\ x_t)$ and the total number of simulated samples N. $C_{sim}(x_m,\ x_t)$ represents the simulated contact count which equals to the number of simulated configurations that satisfy the condition where the distance between x_m and x_t is shorter than 80 nm [4,20].

$$C_{sim}(x_m,\ x_t) = \sum_{k=1}^{N} I\left(d_{mt}^{(k)} \le d_c\right) \quad , \qquad \text{(Supplementary Equation 11)}$$

where $d_{mt}^{(k)}$ is the spatial distance between $x_m^{(k)}$ and $x_t^{(k)}$ in the k-th chain, d_c is the distance threshold of 80 nm.

(2) $P\left(E_t\right)$ is the probability of selecting E_t from all the available ensemble candidates. We determine the polymer ensemble E_t given the positions of previously constructed polymer chains $X_{t-1}^{(1)}, X_{t-1}^{(2)}, \cdots, X_{t-1}^{(N)}$ at the $t-1$ step, therefore, $P\left(E_t\right)$ can be represented as

$$\begin{aligned} P\left(E_t\right) &= P\left(E_t | E_{t-1}\right) \\ &= P\left(X_t^{(1)}, X_t^{(2)}, \cdots, X_t^{(N)} | X_{t-1}^{(1)}, X_{t-1}^{(2)}, \cdots, X_{t-1}^{(N)}\right) \end{aligned}$$

(Supplementary Equation 12)

When we grow the polymer chains, we first list all possible x_t candidates based on the previous ensemble E_{t-1} and filter out the candidates that violate the physical constraints of self-avoiding property. Each chain has a maximum of 50 candidates. We then pool the valid candidates together and pick an initial polymer ensemble from the candidate pool. We assume each valid ensemble candidate has the same probability to be chosen, thus our $P\left(E_t\right)$ is considered to be a constant which is reversely proportional to the number of all possible valid ensembles.

We apply an iterative optimization strategy to find the best polymer ensemble at each step that maximize the probability $P\left(E_t | H_t\right)$. Since $P\left(E_t\right)$ is a constant given the valid candidate pool at each step, our strategy is trying to maximize the first term $P\left(H_t | E_t\right)$. It's as follows

(1) Select an initial polymer ensemble E_t from the candidate pool, calculate the probability $P\left(H_t | E_t\right)$ as mentioned above.

(2) Randomly pick one polymer chain from the initial ensemble and switch it to another valid polymer in the candidate pool, calculate the new probability $P'\left(H_t | E_t\right)$.

(3) If $P'\left(H_t | E_t\right) > P\left(H_t | E_t\right)$, keep the new polymer chain and update the probability,

$$\begin{aligned} P\left(H_t | E_t\right) &= P'\left(H_t | E_t\right) \\ \Delta P &= \frac{P'\left(H_t | E_t\right) - P\left(H_t | E_t\right)}{P\left(H_t | E_t\right)} \end{aligned}$$

(Supplementary Equation 13)

Otherwise, abandon the new chain and keep the old one.

(4) Repeat the step 2-3 until ΔP is convergent, here we use a threshold of 0.0001.

We apply the same optimization procedure at each growing step until the polymer chain grows to the wishful length n.

In silico evaluation of the sequential Bayesian inference framework

To quantitatively evaluate the capability in reconstructing chromatin configurations based on Hi-C measurements of our method, we first construct an original polymer ensemble as the ground truth. This original ensemble consists of 50,000 polymer chains, each of 200 beads. To simulate domain structures, we added an intra-TAD contact weight of 0.3 to each pair of nodes localized between 21 and 60, between 61 and 120, and between 121 and 180. Here contacts are defined as $\leq$ 80 nm Euclidean distance. We added another inter-TAD contact weight of 0.1 to each pair of nodes, with one node localized between 61 and 120 and the other between 121 and 180. We also added a loop contact weight of 0.8 to one loop interaction, with anchors p1 localized in 59 – 63 and p2 in 119 – 123, and another loop interaction with anchors p1 localized in 59 – 63 and p3 in 179 – 183. These contact weights are artificially imposed and are used to provide "driving forces" for TAD formation and loop formation. Further quantitative information can be found in the caption of Supplementary Figure 7.

We then aggregate the contact maps of each single chromatin polymer in our original ensemble and obtain our simulated target "Hi-C" map, from which we then reconstruct another polymer ensemble via our sequential Bayesian inference framework. This reconstructed ensemble consists of 50,000 chromatin polymer chains (Supplementary Figure 7a).

We find beads exhibiting similar distance distributions to other beads, when corresponding beads in the two ensembles are compared. This is demonstrated in Supplementary Figure 7b, which depicts the distance distributions of 5 selected beads

(0-, 40-, 80-, 120-, and 160-th) to all other beads in box plots for the two ensembles. Furthermore, the median fractions of bead pairs between these selected beads and all other beads that are within 80nm are also similar: 4.2% vs. 4.3% for the 0-th node (original vs. reconstructed), 11.9% vs. 12.2% for the 40-th node, 14.9% vs. 14.3% for the 80-th node , 15.5% vs. 15.8% for the 120-th node and 14.2% vs. 13.8% for the 160-th node. We further computed the radius of gyration and end-to-end distance for each simulated polymer chain in both ensembles, and find that there is no statistical difference between the original and the reconstructed ensemble (Supplementary Figure 7c).

We then combine the original and the reconstructed ensembles by randomly selecting 5,000 chains from each ensemble. We further group the mixtures of polymer chains into clusters using hierarchical clustering. This results in 7 structural clusters judged by the silhouette scores (Supplementary Figure 7e). For these 7 clusters (Supplementary Figure 7d), the proportion of each cluster is highly consistent between the original and the reconstructed ensemble (Supplementary Figure 7f). Furthermore, the aggregated contact heat maps of polymer chains from the original ensemble are also strongly similar to those from the reconstructed ensemble for all 7 clusters (Supplementary Figure S7g). In addition, the reconstructed contact map of the full ensemble is also strongly similarity to the original map (r = 0.98, r= 0.86). Our approach can uncover 3-body interaction unit, as shown in Supplementary Figure 7h and 7i.

Our model can also incorporate *a priori* information where three bodies p1, p2, and p3 are known to interact solely as a triplet. Our method can readily generate ensembles that conform to such specifications by knocking-in these simultaneous interactions [17]. This is shown in Supplementary Figure 7j with a toy example of a 4-cell ensemble. Here the simultaneous all-to-all 3-body interaction (p1-p2-p3) is given *a priori* and modeled through explicit knock-in perturbations using our CHROMATIX method [17]. Supplementary Figure 7j shows that the simultaneous 3-body interaction (colored as green, yellow, and purple) is recovered in all four cells. Such *a priori* information may be found, for example, from 3D-FISH studies and can be incorporated to complement Hi-C data using our full suite of computational tools.

For full details, please see "Chromatin-to-chromatin proximity interaction modeling tutorial" available at `https://bitbucket.org/aperezrathke/chr-folder/`.

Comparison with other methods in 3D modeling

Monte Carlo model of ref [4]. In [4], an artificial square-well potential was employed, with the depth, width and location of the potential optimized through curve-fitting to the 5C-data modeled. This optimized potential was then used to generate 5,000 conformations through Metropolis Monte Carlo.

Validation was in the form of agreement with the distributions, mean, and standard deviation of pair-distances of genomic regions measured by FISH. Similar validation is provided in our study (Supplementary Figure 4), where we showed our model can reproduce the distance distributions between Scyl-chrb and distributions of two other pair-interactions.As this study is based on 5C data, no results on comparing simulation with experimental Hi-C data were reported.

MiChroM model. The MiChroM method [24, 25] investigates the energy landscape of chromatin folding. Its aim is to study general folding properties emerging from different factors in the chromatin energy model. A fully specified energy model requires A) additional information along the chromosome. Specifically, A1) beads are assigned to a specific chromatin states; and A2) the locations of loop anchors are specified (such as CTCF sites). Further, there are B) 27 parameters for the physical interactions: interaction energy of beads of different chromatin states; interaction energy of loop anchors; and distance-dependent compaction.

Both A1) and A2) are obtained based on annotation provided by Rao et al [16]. The 27 adjustable parameters are estimated from Hi-C data of chromosome 10. The model is then applied to other chromosomes, in which 2×10^4 steps of MD simulations were carried out to generate chromatin ensembles. Overall, Hi-C contact maps can be reproduced at r of 0.956. In addition, a number of important findings were made with this model, including the likely origin of chromosome territories, phase separation among chromatin types, active genes in the periphery of chromosome territories, and knottedness of chromatin (see also a commentary on this work [26]).

As it was designed to study whole genome folding, the resolution of MiChroM is necessarily limited to 50 kb. Therefore, this model does not provide detailed spatial structures below 50 kb. At a whole chromosome level, our method has similar Pearson correlation coefficient ($r = 0.94$ for *Drosophila* chr X), but at a higher resolution of 5 kb.

MOGEN. The MOGEN method is data-driven and is based on optimization. It employs an empirical objective function designed to satisfy as many high probability restraints as possible, while reducing clashes. Restraints are converted from Hi-C data based on frequency thresholds. Parameters are adjusted so chains can be generated successfully after optimization by gradient ascent. MOGEN was used to generate a set of 500 genomewide chromosome structures using Hi-C data. However, it is unclear how well this approach works at finer base-pair resolutions (e.g. 5 kb).

3C-based Model. The method of Wang et al [12] was developed for analysis of 3C data, in which chromosomes are modeled as connected fragments, whose lengths are determined by restriction enzyme cuts. The idealized energy of each chromatin chain, consisting of terms for excluded volume, bending, and stretching, is combined with 3C measurements under a Bayesian framework, where parameters of a Gaussian-based likelihood function is inferred using the EM algorithm. This method is used to generate an ensemble of yeast genome structures, with the results validated by comparing distance distributions with FISH measurements.

While chromatin structures reported are at fragment resolution, there are other studies of budding yeast, where simulated and measured Hi-C heat maps show excellent correlations [12, 20, 27]. For example, our earlier ensemble models of yeast genome based on a random model similar to that of this study exhibit an excellent whole-genome correlation ($r = 0.82$) at 15 kb resolution [20].

Our method. Our method differs from several other methods [4, 12, 19, 24, 25] in important regards:

Minimal assumptions. Our model is minimalistic. It does not require assigning each bead to a specific chromosome state. There is also no *a priori* assumption on where loop anchor sites are located (e.g. CTCF). Rather, it requires only a small

number of contact probabilities at different loci pairs. These are the specific interactions derived from Hi-C data analysis using our null model. With basic physical assumptions (chromatin fiber density, self-avoiding chains, and Euclidean distance threshold in nm for ligation), there are no adjustable model parameters. The target distribution from which our Hi-C ensembles are constructed is that of self-avoiding chromatin chains satisfying Hi-C derived pairing probabilities.

This minimalistic approach offers a useful advantage, namely, it avoids another level of complexity in deconvolving degeneracy due to a large parameter space, which is in addition to the complexity of deconvolving Hi-C heat maps into an ensemble of 3D chromatin configurations. In models where chromatin beads are assigned to different states and values of binding affinities are required, there are likely many permutations of states and binding affinities that are all consistent with the Hi-C data. Our model avoids this potential problem of limited identifiability.

Deep sampling. Our method also differs from several existing methods in an important aspect. We make use of a well-designed sampling distribution to improve sampling efficiency, which is dynamically adjusted based on the chromatin polymer ensemble at each growing step (Supplementary Figure 2a). The sampled ensemble rigorously follows that of the predefined target distribution, *i.e.*, self-avoiding chromatin chains satisfying Hi-C derived pairing probabilities. This in contrast to several optimization-based method (such as the method of [12]), where each chain is optimized separately through an objective function constructed on single chains (Supplementary Figure 2b).

Our sampling method is built upon significant advancements in sequential importance sampling techniques [8, 17]. As a result, we achieve very deep (*i.e.*, large-scale) sampling. For example, the ensembles we constructed contain 50,000 chromatin configurations, each of 1,000 beads of 2 kb (for loci) or 4,485 beads of 5 kb (for chromosome X), and are independently grown by sequentially adding one bead at a time. With advances in resampling and rejection control techniques far beyond the well-known PERM approach [23], we are able to overcome the well-recognized attrition problem of severe difficulty in growing long SAW chains in confined volume [8, 13].

Our sampling method is also fast. This is shown in Supplementary Figure 8d, where the averaged CPU costs (in minutes) for construction of ensembles for ten regions by specific interactions at different chain length (100–1,000 beads) are provided.

This level of deep sampling likely compares very favorably with previous studies. Under ergodic assumptions, both molecular dynamics (MD) simulations and Markov chain Monte Carlo can generate proper samples after long runs and establishment of convergency. However, achieving convergency is nontrivial, and obtaining decorrelated samples at accessible time scales along simulation trajectories is challenging in both cases. Our ensembles of 5×10^4 chromatin polymers from chain growth are results of better sampling. Compared to MD simulation, our method does not suffer from the issue of in-sufficient simulation time. Compared to MCMC simulations, our method does not suffer from possible slow convergence, which is currently an unsolved problem. In general, our method can generate more quality samples in a shorter time frame, therefore is better equipped at quantifying heterogeneity than those from 2×10^4 steps of MD simulations reported in [25], the 5,000 conformations generated using Metropolis Monte Carlo reported in [4], or typically a few hundred structures obtained using methods based on optimization [19].

Realistic ligation model. Our probabilistic model of ligation is realistic with less assumptions. We assume regions in close spatial proximity below a threshold are available to form Hi-C ligations probabilistically. Hi-C probabilities therefore represent the proportions of polymer chains that satisfy the ligation threshold (Supplementary Figure 2c). In contrast to the approach of [12], where Hi-C contact frequencies are translated to spatial distances via a reverse exponential function under implicit assumptions regarding the rate of chromatin diffusion and how it relates to the mean contact frequency, our model makes no such assumptions (Supplementary Figure 2d).

High resolution. The resolution of our model is high: 2 kb for specific loci, and 5 kb for whole chromosome, which is at the same resolution of many Hi-C studies.

Overall, our method is well-suited to a number of tasks, such as quantitative assessment of chromatin heterogeneity and determination of three-body interactions. It enabled us to reveal the novel insights reported in this study.

Calculation of distance-adjusted Pearson correlation coefficient

To eliminate the effects of genomic distance, we measure the distance-adjusted Pearson correlation r' following ref [22]. Specifically, for each diagonal of the contact matrices, we calculate and subtract the averaged contact frequency at that specific genomic distance for both Hi-C and simulated contact maps. We then calculate the Pearson correlation coefficient after subtraction.

Hierarchical clustering of the single-cell 3D configurations

For each single-cell chromatin configuration, we calculate the spatial distances of each pair of beads and generate a ligation matrix, which only contains 1 (spatial distance $\leq$ 80 nm) or 0 (spatial distance $>$ 80 nm). We randomly pick 5,000 configurations from each polymer ensemble of the three cell types and concatenate the ligation matrix into a one-dimensional ligation vector for each single-cell conformation. Similar to the clustering of specific interactions, we use a Hierarchical clustering (Agglomerative Clustering) [9] program to categorize these 15,000 single-cell ligation vectors of all three cell types into 5 clusters. The number of clusters is determined according to the Silhouette scores.

Comparison of modeled and measured single-cell conformations

We generated 10,000 single-cell conformations of the locus chr3R:12.20-12.90 Mb using Hi-C data of S2R+ cells. The resulting distance maps are compared pixel-wise with that depicted in Mateo et al [30], where 19,103 cells were measured, and two depicted. Despite the gap regions in reported results in Mateo et al, which makes direct comparison difficult, two single-cell conformations we modeled have overall excellent agreement with the two conformations depicted in Mateo et al, with $R = 0.75$ and 0.79, respectively.

Identification of single-cell domain boundaries and TAD-like structures

With the chromatin polymers generated, we adopt a similar method to define the domain boundaries in single cells as that described in [2]. Briefly, we calculate the Euclidean distance of each pair of beads and generated spatial-distance matrices for each chromatin conformation. Before identification of the domain boundaries, each spatial-distance matrix is subject to Gaussian filtering using a window of 25 bins for noise reduction. For each bead i and a window size w, we compute the average distance of all bead pairs with both beads localized between $i - w$ and i as a_1, the average distance of all bead pairs with both beads localized between $i+1$ and $i+w+1$ as a_2, and the average distance of all bead pairs with one bead localized between $i-w$ and i, and another between $i+1$ and $i+w+1$ as b. We define the boundary strength as the ratio of b to the geometric mean of a_1 and a_2. We draw a curve of all boundary strengths along the linear genomic positions in each cell and search for local maxima. All local maxima above a threshold of s are chosen to be the single-cell domain boundaries. After testing various w and s, we find the selected domain boundaries are more consistent with the heat maps of spatial-distance matrices when $w = 50$ and $s = 2.2$.

After identification of domain boundaries, the consecutive region between each pair of adjacent boundaries are identified as TAD candidates. For each TAD candidate, we calculated the average spatial distances within the TAD. We assign a p-value to each candidate by calculating the proportion of random chromatin polymers where the average spatial distance of the corresponding TAD region is lower than that of the TAD candidate. TAD candidates with BH-FDR below 0.05 are selected to be the TAD-like structures.

Similar to earlier findings [13], many chromatin configurations in a control random ensemble of chr2L: 11.0–12.0 Mb also exhibit TAD-like structures: 23.1% are found to maintain at least one TAD-like structure. However, this is significantly less compared to 54.4% of chromatin configurations in embryos at pre-MBT stages (cycles 9–13)

(Figure 5a). Furthermore, the TAD boundaries are distributed very evenly in the random ensemble, in contrast to cells at pre-MBT and later stages (Supplementary Figure 11a). These results suggest that while TAD-like structures may form in random chromatin configurations, other biological factors beyond fluctuation play significant roles during TAD formation in early embryos.

Comparison with Hug et al.

We compared our modeled ensemble of single-cell conformations for the region of chr2L:4.5-6.4 Mb with the study of Hug et al [29]. Supplementary Figure 11d shows the boundary probabilities estimated from our ensemble (see third track), which are constructed from the Hi-C data of embryos at cycles 9–13 [18]. Overall, our estimated boundary probabilities agree well with the insulation scores and RNA Pol II ChIP-Seq signal derived from Hug et al [29].

Evaluation of boundary strength by Directionality Index

We also evaluated the boundary strength directly using population Hi-C contact matrices. We calculated the Directionality Index (DI) for each 2 kb bin following ref [28]. DI is calculated as:

$$DI = (\frac{f_{down} - f_{up}}{|f_{down} - f_{up}|})(\frac{(f_{up} - f_{exp})^2}{f_{exp}} + \frac{(f_{down} - f_{exp})^2}{f_{exp}}),$$

(Supplementary Equation 14)

where f_{up} is the contact frequency between the given 2 kb bin and the upstream 100 kb region; f_{down} is the contact frequency between the same bin and the downstream 100 kb region; f_{exp} is the expected contact frequency, which equals to $\frac{f_{up}+f_{down}}{2}$. We find that embryos at post-MBT (stages 5–8) and S2R+ show strong DI signals, which indicates clear TAD structures with sharp boundaries. In contrast, DI signals are weak or non-existent in early embryos at pre-MBT stages (cycles 9–13) (Supplementary Figure 11b), indicating lack of TAD structures as judged from population Hi-C contact matrix (Figure 5a).

Supplementary References

[1] Yoav Benjamini, Daniel Yekutieli, et al. The control of the false discovery rate in multiple testing under dependency. *Annals of Statistics*, 29(4):1165–1188, 2001.

[2] Bogdan Bintu, Leslie J Mateo, Jun-Han Su, Nicholas A Sinnott-Armstrong, Mirae Parker, Seon Kinrot, Kei Yamaya, Alistair N Boettiger, and Xiaowei Zhuang. Super-resolution chromatin tracing reveals domains and cooperative interactions in single cells. *Science*, 362(6413), 2018.

[3] Yad Ghavi-Helm, Felix A Klein, Tibor Pakozdi, Lucia Ciglar, Daan Noordermeer, Wolfgang Huber, and Eileen EM Furlong. Enhancer loops appear stable during development and are associated with paused polymerase. *Nature*, 512(7512):96–100, 2014.

[4] Luca Giorgetti, Rafael Galupa, Elphège P Nora, Tristan Piolot, France Lam, Job Dekker, Guido Tiana, and Edith Heard. Predictive polymer modeling reveals coupled fluctuations in chromosome conformation and transcription. *Cell*, 157(4):950–963, 2014.

[5] Gamze Gürsoy, Yun Xu, Amy L Kenter, and Jie Liang. Computational construction of 3d chromatin ensembles and prediction of functional interactions of alpha-globin locus from 5c data. *Nucleic Acids Research*, 45(20):11547–11558, 2017.

[6] Peter V Kharchenko, Artyom A Alekseyenko, Yuri B Schwartz, Aki Minoda, Nicole C Riddle, Jason Ernst, Peter J Sabo, Erica Larschan, Andrey A Gorchakov, Tingting Gu, et al. Comprehensive analysis of the chromatin landscape in drosophila melanogaster. *Nature*, 471(7339):480–485, 2011.

[7] Ariel Kleiner, Ameet Talwalkar, Purnamrita Sarkar, and Michael I Jordan. A scalable bootstrap for massive data. *Journal of the Royal Statistical Society: Series B: Statistical Methodology*, pages 795–816, 2014.

[8] Jun S Liu. *Monte Carlo strategies in scientific computing.* Springer Science & Business Media, 2008.

[9] Fabian Pedregosa, Gaël Varoquaux, Alexandre Gramfort, Vincent Michel, Bertrand Thirion, Olivier Grisel, Mathieu Blondel, Peter Prettenhofer, Ron Weiss, Vincent Dubourg, et al. Scikit-learn: Machine learning in python. *Journal of Machine Learning Research*, 12(Oct):2825–2830, 2011.

[10] Graham Upton and Ian Cook. *Understanding statistics.* Oxford University Press, 1996.

[11] Nelle Varoquaux, Ferhat Ay, William Stafford Noble, and Jean-Philippe Vert. A statistical approach for inferring the 3d structure of the genome. *Bioinformatics*, 30(12):i26–i33, 2014.

[12] Siyu Wang, Jinbo Xu, and Jianyang Zeng. Inferential modeling of 3d chromatin structure. *Nucleic Acids Research*, 43(8):e54–e54, 2015.

[13] Gamze Gürsoy, Yun Xu, Amy L Kenter, and Jie Liang. Spatial confinement is a major determinant of the folding landscape of human chromosomes. *Nucleic Acids Research*, 42(13):8223–8230, 2014.

[14] Ferhat Ay, Timothy L Bailey, and William Stafford Noble. Statistical confidence estimation for hi-c data reveals regulatory chromatin contacts. *Genome Research*, 24(6):999–1011, 2014.

[15] Borbala Mifsud, Inigo Martincorena, Elodie Darbo, Robert Sugar, Stefan Schoenfelder, Peter Fraser, and Nicholas M Luscombe. Gothic, a probabilistic model to resolve complex biases and to identify real interactions in hi-c data. *PLoS One*, 12(4):e0174744, 2017.

[16] Suhas SP Rao, Miriam H Huntley, Neva C Durand, Elena K Stamenova, Ivan D Bochkov, James T Robinson, Adrian L Sanborn, Ido Machol, Arina D Omer, Eric S Lander, et al. A 3d map of the human genome at kilobase resolution reveals principles of chromatin looping. *Cell*, 159(7):1665–1680, 2014.

[17] Alan Perez-Rathke, Qiu Sun, Boshen Wang, Valentina Boeva, Zhifeng Shao, and Jie Liang. Chromatix: computing the functional landscape of many-body chromatin interactions in transcriptionally active loci from deconvolved single cells. *Genome Biology*, 21(1):1–17, 2020.

[18] Yuki Ogiyama, Bernd Schuettengruber, Giorgio L Papadopoulos, Jia-Ming Chang, and Giacomo Cavalli. Polycomb-dependent chromatin looping contributes to gene silencing during drosophila development. *Molecular Cell*, 71(1):73–88, 2018.

[19] Tuan Trieu and Jianlin Cheng. Mogen: a tool for reconstructing 3d models of genomes from chromosomal conformation capturing data. *Bioinformatics*, 32(9):1286–1292, 2016.

[20] Gamze Gürsoy, Yun Xu, Amy L Kenter, and Jie Liang. Computational construction of 3d chromatin ensembles and prediction of functional interactions of alpha-globin locus from 5c data. *Nucleic Acids Research*, 45(20):11547–11558, 2017.

[21] Andrew S Belmont. Large-scale chromatin organization: the good, the surprising, and the still perplexing. *Current Opinion in Cell Biology*, 26:69–78, 2014.

[22] Simona Bianco, Darío G Lupiáñez, Andrea M Chiariello, Carlo Annunziatella, Katerina Kraft, Robert Schöpflin, Lars Wittler, Guillaume Andrey, Martin Vingron, Ana Pombo, et al. Polymer physics predicts the effects of structural variants on chromatin architecture. *Nature Genetics*, 50(5):662–667, 2018.

[23] Hsiao-Ping Hsu and Peter Grassberger. A review of monte carlo simulations of polymers with perm. *Journal of Statistical Physics*, 144(3):597, 2011.

[24] Bin Zhang and Peter G Wolynes. Topology, structures, and energy landscapes of human chromosomes. *Proceedings of the National Academy of Sciences*, 112(19):6062–6067, 2015.

[25] Michele Di Pierro, Bin Zhang, Erez Lieberman Aiden, Peter G Wolynes, and José N Onuchic. Transferable model for chromosome architecture. *Proceedings of the National Academy of Sciences*, 113(43):12168–12173, 2016.

[26] Gamze Gürsoy and Jie Liang. Three-dimensional chromosome structures from energy landscape. *Proceedings of the National Academy of Sciences*, 113(43):11991–11993, 2016.

[27] Harianto Tjong, Ke Gong, Lin Chen, and Frank Alber. Physical tethering and volume exclusion determine higher-order genome organization in budding yeast. *Genome Research*, 22(7):1295–1305, 2012.

[28] Jesse R Dixon, Siddarth Selvaraj, Feng Yue, Audrey Kim, Yan Li, Yin Shen, Ming Hu, Jun S Liu, and Bing Ren. Topological domains in mammalian genomes identified by analysis of chromatin interactions. *Nature*, 485(7398):376–380, 2012.

[29] Clemens B Hug, Alexis G Grimaldi, Kai Kruse, and Juan M Vaquerizas. Chromatin architecture emerges during zygotic genome activation independent of transcription. *Cell*, 169(2):216–228, 2017.

[30] Leslie J Mateo, Sedona E Murphy, Antonina Hafner, Isaac S Cinquini, Carly A Walker, and Alistair N Boettiger. Visualizing dna folding and rna in embryos at single-cell resolution. *Nature*, 568(7750):49–54, 2019.
