# Supplementary material for: High-resolution single-cell 3D-models of chromatin ensembles during Drosophila embryogenesis
Source: Nat Commun. 2021 Jan 8;12:205. doi: 10.1038/s41467-020-20490-9 (PMC7794469; doi:10.1038/s41467-020-20490-9)
Supplement: Supplementary file 3 — Reporting Summary [file 41467_2020_20490_MOESM3_ESM.pdf]

nature research

Corresponding author(s): Zhifeng Shao & Jie Liang

Last updated by author(s): Sep 10, 2020

Reporting Summary

Nature Research wishes to improve the reproducibility of the work that we publish. This form provides structure for consistency and transparency in reporting. For further information on Nature Research policies, see Authors & Referees and the Editorial Policy Checklist .

Statistics

For all statistical analyses, confirm that the following items are present in the figure legend, table legend, main text, or Methods section.

n/a	Confirmed	
☐	☒	The exact sample size (n) for each experimental group/condition, given as a discrete number and unit of measurement
☐	☒	A statement on whether measurements were taken from distinct samples or whether the same sample was measured repeatedly
☐	☒	The statistical test(s) used AND whether they are one- or two-sided<br>*Only common tests should be described solely by name; describe more complex techniques in the Methods section.*
☒	☐	A description of all covariates tested
☐	☒	A description of any assumptions or corrections, such as tests of normality and adjustment for multiple comparisons
☐	☒	A full description of the statistical parameters including central tendency (e.g. means) or other basic estimates (e.g. regression coefficient) AND variation (e.g. standard deviation) or associated estimates of uncertainty (e.g. confidence intervals)
☐	☒	For null hypothesis testing, the test statistic (e.g. F, t, r) with confidence intervals, effect sizes, degrees of freedom and P value noted<br>*Give P values as exact values whenever suitable.*
☐	☒	For Bayesian analysis, information on the choice of priors and Markov chain Monte Carlo settings
☒	☐	For hierarchical and complex designs, identification of the appropriate level for tests and full reporting of outcomes
☐	☒	Estimates of effect sizes (e.g. Cohen's d, Pearson's r), indicating how they were calculated

Our web collection on statistics for biologists contains articles on many of the points above.

Software and code

Policy information about availability of computer code

Data collection	We downloaded the raw Hi-C data from the GEO database (https://www.ncbi.nlm.nih.gov/geo/) with accession number GSE103625 (embryos of Post-MBT and Pre-MBT) and GSE101317 (S2R+).
Data analysis	We first map Hi-C data to Drosophila dm3 reference genome using Bowtie2 (v 2.2.9). After filtering out invalid Hi-C reads, we generate Hi-C contact maps at 2/5 kb resolution. Hi-C matrices are normalized using ICE from hiclib (https://bitbucket.org/mirnylab/hiclib). We construct physical null model via Fractal Monte Carlo (https://bitbucket.org/aperezrathke/chr-folder). We bootstrap our random ensembles and Hi-C interactions with BH-FDR below 0.01 are selected to be our specific interactions. We cluster the specific interactions into 6 types based on the ChIP-chip signals downloaded from modENCODE(Supplementary Table S2) using Agglomerative clustering from scikit-learn (v 0.21.2). Specific interactions are used for construction of single-cell 3D chromatin conformations under the framework of sequential Bayesian inference (https://github.com.qiusun0215/sBIF).<br>We compared our specific interactions with those identified by FitHi-C (v 2.0.7), GOTHiC (v 1.22.0), and HICCUPS (v 1.14.08).

For manuscripts utilizing custom algorithms or software that are central to the research but not yet described in published literature, software must be made available to editors/reviewers. We strongly encourage code deposition in a community repository (e.g. GitHub). See the Nature Research guidelines for submitting code & software for further information.

Data

Policy information about availability of data

All manuscripts must include a data availability statement. This statement should provide the following information, where applicable:

- Accession codes, unique identifiers, or web links for publicly available datasets
- A list of figures that have associated raw data
- A description of any restrictions on data availability

Hi-C data are downloaded from GEO database (embryos at cycles 9-13 and stages 5-8 from GSE103625, S2R+ from GSE101317). ChIP-chip datasets for clustering are downloaded from modENCODE database with IDs listed in Supplementary Table 2.

Expression level of gene Scyl (top) and chrb (bottom) during Drosophila embryogenesis are downloaded from Flybase (https://flybase.org/).
The source data underlying Figures 2c-d, 3d, 4d, 5g and Supplementary Figures 3d, 4b, 5, 7c, 7h, 8c, 9c are provided as a Source Data file.

Field-specific reporting

Please select the one below that is the best fit for your research. If you are not sure, read the appropriate sections before making your selection.

- [x] Life sciences
- [ ] Behavioural & social sciences
- [ ] Ecological, evolutionary & environmental sciences

For a reference copy of the document with all sections, see nature.com/documents/nr-reporting-summary-flat.pdf

Life sciences study design

All studies must disclose on these points even when the disclosure is negative.

Sample size	For each simulation, we generate 50,000 single-chain configurations, this sample size is determined based on the Pearson Correlation Coefficient between our simulated Hi-C contact map and the original Hi-C map. Pearson's r are all above 0.9 at this sample size.
Data exclusions	During the null model construction, we removed samples with extreme high weights by a 1.5*IQR standard which is commonly used during data analysis. Please see more details in the Supplementary Methods.
Replication	We constructed chromatin structures in ten different regions and at two different resolutions (2kb or 5kb) using our method, all simulations are successful and have high correlations with the corresponding Hi-C contact maps.
Randomization	Not relevant, sequencing data of known cell types are all downloaded from the public database
Blinding	Not relevant, sequencing data of known cell types are all downloaded from the public database

Reporting for specific materials, systems and methods

We require information from authors about some types of materials, experimental systems and methods used in many studies. Here, indicate whether each material, system or method listed is relevant to your study. If you are not sure if a list item applies to your research, read the appropriate section before selecting a response.

Materials & experimental systems

n/a	Involved in the study	
x		Antibodies
	x	Eukaryotic cell lines
x		Palaeontology
x		Animals and other organisms
x		Human research participants
x		Clinical data

Methods

n/a	Involved in the study	
x		ChIP-seq
x		Flow cytometry
x		MRI-based neuroimaging

Eukaryotic cell lines

Policy information about cell lines

Cell line source(s)	Drosophila embryos at cycles 9-13 and stages 5-8 (GSE103625) and S2R+ (GSE101317)
Authentication	N/A
Mycoplasma contamination	N/A
Commonly misidentified lines (See ICLAC register)	N/A
